# Supplementary material for: Genotype-free demultiplexing of pooled single-cell RNA-seq
Source: Genome Biol. 2019 Dec 19;20:290. doi: 10.1186/s13059-019-1852-7 (PMC6921391; doi:10.1186/s13059-019-1852-7)
Supplement: Supplementary file 1 — Additional file 1 Figure S1. Illustration of presence absence matrices calculated on pooled and hashtagged scRNA-seq datasets. Figure S2. Illustration of presence absence matrices calculated on pooled fibroblast scRNA-seq datasets. [file 13059_2019_1852_MOESM1_ESM.pdf]

hashtag	scSplit								vcf							
	0	1	2	3	4	5	6	7	D	E	F	A	G	B	H	C
10:106022789	0	1	1	0	1	0	1	1	0	0	1	1	1	1	1	0
11:252649	0	0	1	0	0	0	0	1	0	0	0	0	1	0	1	0
12:7053149	1	1	1	0	0	1	1	0	1	1	0	1	1	1	0	0
19:50168871	0	1	1	1	1	0	0	1	0	0	1	1	1	0	1	1
1:151374025	0	0	1	0	1	1	0	0	0	1	1	0	1	0	0	0
21:30408670	0	0	0	1	1	0	1	0	0	0	1	0	0	1	0	1
3:69153854	0	0	0	0	0	1	1	1	0	1	0	0	0	1	1	0
5:96031569	1	0	1	0	0	0	1	1	1	0	0	0	1	1	1	0

Figure S1 Illustration of presence absence matrices calculated on pooled and hashtagged scRNA-seq datasets. Within each rectangle we showed the P/A genotype for each sample caclulated on scRNA-seq data using scSplit, and the corresponding P/A genotype calculated from matched genotype files. The mapping of scRNA-seq sample to genotype sample was indicated by colors.

mix1	scSplit							imputed vcf						
	1	2	3	4	5	6	7	11	28	53	110	181	195	326
10:135233840	1	0	0	1	0	0	1	0	1	0	0	1	1	0
13:111293915	0	0	1	0	0	0	1	0	0	0	0	0	1	1
16:15131962	1	1	1	1	1	1	0	1	1	1	1	1	0	1
17:46103760	0	0	1	0	1	0	1	0	0	0	1	0	1	1
1:151374025	0	1	0	0	1	1	1	1	0	1	1	0	1	0
4:166264119	0	0	1	0	1	1	0	1	0	0	1	0	0	1
4:1694809	0	1	0	1	0	0	1	0	0	1	0	1	1	0

mix2	scSplit								imputed vcf							
	1	2	3	4	5	6	7	8	104	114	137	145	161	184	212	253
11:67353579	0	0	1	1	0	1	0	1	0	1	0	0	1	1	0	1
12:57924050	0	0	0	1	0	0	1	1	0	1	0	1	0	1	0	0
14:51372103	1	0	1	0	1	0	0	1	0	1	1	0	0	0	1	1
14:75408789	0	1	1	1	0	0	0	0	1	0	0	0	0	1	0	1
17:66528778	1	0	0	1	0	0	1	0	0	0	1	1	0	1	0	0
19:6751293	0	0	0	0	0	1	1	0	0	0	0	1	1	0	0	0
2:228222293	0	0	1	1	1	0	1	0	0	0	0	1	0	1	1	1
3:66428282	0	0	0	0	1	1	0	1	0	1	0	0	1	0	1	0

mix3	scSplit								imputed vcf							
	0	1	2	3	4	5	6	7	77	78	2	31	169	43	124	320
14:71051088	0	0	0	1	0	0	1	0	0	1	0	0	0	0	1	0
19:49379167	0	0	1	0	0	0	1	0	0	1	0	0	0	1	0	0
19:51301230	0	0	0	0	1	1	1	0	1	1	0	0	1	0	0	0
1:162753835	1	0	0	0	0	0	0	0	0	0	0	1	0	0	0	0
1:33789382	1	0	0	0	0	0	1	1	0	1	1	1	0	0	0	0
2:99978048	0	0	1	0	1	1	0	1	1	0	1	0	1	1	0	0
4:10076860	0	1	0	0	0	1	1	1	1	1	1	0	0	0	0	1
9:35660901	0	1	0	0	1	0	1	0	0	1	0	0	1	0	0	1

mix4	scSplit								imputed vcf							
	0	1	2	3	4	6	7	8	48	173	147	171	205	192	324	76
15:49776957	0	1	1	0	1	0	0	0	0	0	1	0	0	1	1	0
19:19287928	0	1	0	0	0	1	1	1	1	0	0	1	0	1	0	1
19:35660752	1	1	1	1	0	1	0	1	1	1	1	0	1	1	0	1
1:68170255	0	0	0	1	1	1	1	0	1	0	0	1	1	0	1	0
2:120059921	1	1	0	0	0	1	0	0	1	1	0	0	0	1	0	0
3:52728804	0	1	0	1	1	1	0	0	1	0	0	0	1	1	1	0
7:140706031	1	0	0	1	0	0	0	0	0	1	0	0	1	0	0	0
8:11700101	0	1	1	0	1	0	0	1	0	0	1	0	0	1	1	1

mix5	scSplit							imputed vcf						
	0	1	2	3	4	5	7	88	176	235	193	265	5	39
10:95104365	1	0	1	0	0	0	0	1	0	0	0	0	0	1
12:121675714	0	1	0	0	1	0	0	0	0	1	1	0	0	0
13:28241287	1	1	0	1	0	0	0	0	0	1	0	0	1	1
2:101622728	0	0	0	1	1	0	0	0	0	0	1	0	1	0
5:9548043	0	0	0	1	0	1	1	0	1	0	0	1	1	0
7:100958270	1	1	0	0	0	1	0	0	0	1	0	1	0	1
7:66121623	0	0	0	0	1	0	1	0	1	0	1	0	0	0

Figure S2 Illustration of presence absence matrices calculated on pooled fibroblast scRNA-seq datasets. Within each rectangle we show the P/A genotype for each sample caclulate on scRNA-seq data using scSpit, and the corresponding P/A genotype calculated from matched genotype files. The mapping of scRNA-seq sample to genotype sample was indicated by colors.
