# Supplementary material for: Genotype-free demultiplexing of pooled single-cell RNA-seq
Source: Genome Biol. 2019 Dec 19;20:290. doi: 10.1186/s13059-019-1852-7 (PMC6921391; doi:10.1186/s13059-019-1852-7)
Supplement: Supplementary file 2 — Additional file 2 Table S1. Accuracy of alternative allele Presence/Absence genotypes built from scSplit/demuxlet clusters compared with that from sample genotyping, based on Hashtag scRNA-seq dataset. Table S2. Simulation using full sibling genotypes from UK Biobank shows scSplit can work for very closely related pooled samples. [file 13059_2019_1852_MOESM2_ESM.pdf]

**Genotyping**	Accuracy	NA
scSplit	0.712	77%
demuxlet	0.725	79%

Table S1 Accuracy of alternative allele Presence/Absence genotypes built from scSplit/demuxlet clusters compared with that from sample genotyping, based on Hashtag scRNA-seq dataset. Accuracy is the proportion of correctly generated genotypes among all non-NA genotypes; NA is the percentage of NA genotypes among all possible elements (shared SNVs x samples)

**Full sieblings**	pair 1	pair 2	pair 3
Mixed samples	2	2	2
Number of cells	12,383	12,383	12,383
Reads per cell	4,973	4,973	4,973
Informative SNVs	258,077	258,077	258,077
Assigning cells	1h30m	1h30m	1h30m
Singlet TPR	0.93	0.82	0.87
Singlet FDR	5E-4	2E-3	9E-4
Doublet TPR	0.997	0.997	1
Doublet FDR	3E-3	5E-3	3E-3
Cohen's Kappa	1	1	1

Table S2 Simulation using full sibling genotypes from UK Biobank shows scSplit can work for very closely related pooled samples
