# Supplementary material for: Genotype-free demultiplexing of pooled single-cell RNA-seq
Source: Genome Biol. 2019 Dec 19;20:290. doi: 10.1186/s13059-019-1852-7 (PMC6921391; doi:10.1186/s13059-019-1852-7)
Supplement: Supplementary file 3 — Additional file 3 Review history. [file 13059_2019_1852_MOESM3_ESM.docx]

Review History

**First round of review**

**Reviewer 1**

**Were you able to assess all statistics in the manuscript, including the appropriateness of statistical tests used?**

Yes: I was able to access

**Were you able to directly test the methods?**

Yes

**Comments to author:**

Here, Xu et al., present scSplit, a bioinformatic tool that enables sample demultiplexing of mixed scRNA-seq that does not require prior genotype knowledge. scSplit uses existing tools to enable the identification of putative variant sites, based on SNVs alone, from scRNA-seq data, followed by modeling allelic counts and assigning cells to clusters via an expectation-maximization framework. This approach has been thoroughly benchmarked against Demuxlet, a current gold-standard genomic-based demultiplexing tool, where scSplit performs better. Moreover, unlike other current approaches, prior genotyping or experimental labeling of cells is not required, placing scSplit as a state-of-the-art demultiplexing tool that I expect will be widely adopted. Overall, this is an impressive and well-executed approach. I have the following specific comments, mainly concerning the exploration of scSplit limitations, which would be helpful for users:

- I appreciated the care taken by the authors to model doublets - this is an important consideration. In this context, will it be possible to 'overload' cells in a run (as has previously been suggested as a cost-saving measure), leading to high doublet rates and later relying on scSplit to identify doublets. Is there an upper-limit of doublet detection at which point demultiplexing would break down?

- The authors state that "Common SNPs of a population (for example The international Genome sample resource (IGSR)) could be used to filter the found SNVs, and minimize potential noises from non-common SNPs.". Have the authors tried this? Is it a better approach?

- Do the authors have any suggestions for how deep each single-cell should be sequenced for demultiplexing to be reliable? The authors might consider downsampling the seq data to demonstrate reliability with sparser datasets.

- Is there an upper limit for how many samples can be demultiplexed? How different do individual genotypes need to be?

- Providing an option for users with existing genotype information is excellent, as it the tutorial on GitHub in general.

- The authors might consider using Cohen's Kappa as a metric when benchmarking scSplit against Demuxlet and Cell Hashing.

- At the end of the manuscript, the authors state that, "Our method and software is broadly applicable in a variety of biological and medical research areas, including but not limited to, distinguishing mixed infections, delineating tumor sub-clones and sequence analysis in non-model organisms." This is not demonstrated and since the current version of scSplit assumes that the number of mixed samples is known, this statement could be toned-down.

Minor comments:

- The authors dive straight into the method and provide key rationale after. This would have been better-placed upfront.

- On GitHub, it would be nice to see some information on expected runtime.

**Reviewer 2**

**Were you able to assess all statistics in the manuscript, including the appropriateness of statistical tests used?**

Yes: The authors implemented an EM algorithm to iteratively optimize an allele fraction model, in order to assign cells to specific clusters. The algorithm was validated on simulated data and multiple set of experimental data.

**Were you able to directly test the methods?**

No

**Comments to author:**

In this manuscript, the authors reported a computational method, scSplit, for demultiplexing single-cell RNA-seq data on pool cells of different genetic backgrounds. As single-cell RNA-seq is getting more and more widely adopted, there is a growing need to analyze multiple samples of different genetic background at once. This has one advantage of reducing batch difference, with an additional benefit of removing doublets. The first method of this sort was published by Kang et al NBT 2017, which relies on SNPs in human population. However, that method requires extra experimental steps (and the associated cost) to genotype all samples. scSplit was developed to specifically address this limitation, based on the idea that one can identify the genetic variants from the scRNA-seq data directly, without extra experiments. This is a rather obvious idea, but implementation is not trivial, because there are many caveats in deriving genotypes from RNA-seq data well documented in the literature. The authors did a very thorough job in implementing the idea, and performed extensive validation and benchmarking, using simulated data as well as real scRNA-seq data . The performance appears to be as good as or in some aspect even better than the Demuxlet method. The authors have made scSplit openly available on github. It will likely be adopted widely by the community, and lead to broad impacts, especially since scSplit can be used on any organism as long as the genome reference is available.

I do have two questions that the authors can hopefully clarify.

(1) For the simulated data set in Table 1, the reads per cell is relatively low, compared with the real experimental data in Table 4. It is surprising that the informative SNVs identified were more or less at a similar level. Is it possible to call 34,116 SNVs with 1,041 reads per cell? Also can the authors comment on the impact of sequencing depth on the accuracy of demultiplexing? When scSplit would stop working?

(2) For the last Results section on batch effects, the conclusion was made based on visual comparison. Can the authors come up with a quantitative way to determine how big or small the batch effects are?

**Authors Response**

**Point-by-point responses to the reviewers’ comments:**

We would like to take this opportunity to address all of the comments from the reviewers as following.

Reviewer #1:

- I appreciated the care taken by the authors to model doublets - this is an important consideration. In this context, will it be possible to 'overload' cells in a run (as has previously been suggested as a cost-saving measure), leading to high doublet rates and later relying on scSplit to identify doublets. Is there an upper-limit of doublet detection at which point demultiplexing would break down?

*As the reviewer pointed out, when cells are ‘overloaded’ in scRNA-seq, more doublets could be generated, and our purpose would be to keep singlet FDR low. We have tested our tool on datasets with different doublet ratios, and it seems the singlet FDR increased when the doublet ratio increased (FDR=0.02 for merged dataset with 5% doublets in table 2, FDR=0.07 for fibroblast dataset with 5% doublets in table 5 and FDR=0.13 for hashtag dataset with 17% doublets as in table 3). Thus the tolerance level on singlet FDR required by the user would define the acceptable experimental degree of cell overloading.*

- The authors state that "Common SNPs of a population (for example The international Genome sample resource (IGSR)) could be used to filter the found SNVs, and minimize potential noises from non-common SNPs.". Have the authors tried this? Is it a better approach?

*We found that common SNPs did improve the performance for some datasets, e.g. the hashtag dataset (table 3) and 8-mix PBMC dataset (table 4). We believe common SNPs helped remove noise contained in these datasets, so we marked it as “highly recommended” in the guideline on our Github page.*

*We described the use of common SNPs filtering in the Methods section as below:*

*...*

*“Next, we tested scSplit on a published scRNA-seq dataset (GSE108313) which used cell-hashing technology to mark samples of the cells before multiplexing. We ran through the scSplit pipeline with the SNVs filtered by common SNVs provided on The International Genome Sample Resource (IGSR)”*

*…*

*“We ran scSplit with common SNVs filtering on published data from the demuxlet paper. By taking demuxlet predictions as ground truth, we achieved high singlet TPR (0.80), although the doublet prediction of the two tools were quite distinct to each other”*

- Do the authors have any suggestions for how deep each single-cell should be sequenced for demultiplexing to be reliable? The authors might consider downsampling the seq data to demonstrate reliability with sparser datasets.

*Our results on different datasets covered a wide range of sequencing depth from the lowest 5,835 rpc in hashtag dataset (table 3), to 14,495 rpc in stromal cell dataset (table 2), to between 16,000 and 22,000 rpc in fibroblast datasets (table 5, Mix2 - Mix5), to 22,757 rpc in PBMC dataset (table 4), and to 86,148 rpc in one of the fibroblast datasets (table 5, Mix1). The good performance even in the relatively sparse hashtag dataset indicated the robustness of the approach for datasets with different sequencing depths.*

*We also downsampled the stromal dataset, which was reported in table 2, to 2,800 rpc to test performance under very low sequencing depth and the result was also acceptable with overall TPR=0.91 and FDR=0.03. We have now included this in the result section “scSplit performed similarly well to demuxlet in demultiplexing merged individually-sequenced three stromal samples”.*

- Is there an upper limit for how many samples can be demultiplexed? How different do individual genotypes need to be?

*We ran additional tests on simulated 12-, 16-, 24-, and 32-mixed samples, and the results were consistently good as 2-, 3-, 4-, and 8-mixed samples. We did observe a minimal drop of TPR in 32-mixed samples, but it was still very high (0.96).*

*To test the limit of our tool on genotype difference, we downloaded three pairs of full sibling genotypes from UK Biobank and simulated pooled samples by mixing one pair at a time, and the average overall TPR of demultiplexing was beyond 0.87. We have reported these new results in “Simulation run showed high accuracy and efficiency of scSplit” of results section in the updated manuscript.*

- Providing an option for users with existing genotype information is excellent, as it the tutorial on GitHub in general.

*Thanks for the comment!*

- The authors might consider using Cohen's Kappa as a metric when benchmarking scSplit against Demuxlet and Cell Hashing.

*We have calculated and updated all tables with Cohen’s Kappa index.*

- At the end of the manuscript, the authors state that, "Our method and software is broadly applicable in a variety of biological and medical research areas, including but not limited to, distinguishing mixed infections, delineating tumor sub-clones and sequence analysis in non-model organisms." This is not demonstrated and since the current version of scSplit assumes that the number of mixed samples is known, this statement could be toned-down.

*We have changed the conclusion to include our future plan to implement autodetection of sample number, which will be helpful in applying to the mentioned biological areas. Here is the revised text in the “Conclusions”:*

*“scSplit is an accurate, fast and computationally efficient method with which to conduct demultiplexing of individual cells from pooled samples of scRNA-seq. In the near future, we plan to enable auto-detection of the mixed sample number, which will help to broaden the application of our tool to more biological and medical research areas, including but not limited to, distinguishing mixed infections, delineating tumor sub-clones and sequence analysis in non-model organisms.”*

- The authors dive straight into the method and provide key rationale after. This would have been better-placed upfront.

*Thanks for the comment and we have moved the Methods section to the end of the manuscript, which is consistent with the required format by Genome Biology - Method.*

- On GitHub, it would be nice to see some information on expected runtime.

*We have added an estimation of runtime into README 3(d) and 4(e) on Github.*

Reviewer #2:

- For the simulated data set in Table 1, the reads per cell is relatively low, compared with the real experimental data in Table 4. It is surprising that the informative SNVs identified were more or less at a similar level. Is it possible to call 34,116 SNVs with 1,041 reads per cell? Also can the authors comment on the impact of sequencing depth on the accuracy of demultiplexing? When scSplit would stop working?

*Thanks for pointing this out and we should have been clear on this that 1,041 reads per cell was for the reads overlapping with the SNVs we used for simulation. The allele matrices were simulated for 12,383 cells using a 32-sample VCF and a template BAM file with 61,576,853 reads, which was actually 4,973 rpc. Among the 61,576,853 reads, 12,889,279 overlapped with the 34,116 SNVs from the VCF file (not called from the BAM file) for simulation, so the reads per cell that we used in building the matrices was counted as 1,041. To avoid confusion, we have replaced 1,041 rpc with 4,973 rpc in table 1 and added relevant information into the “Data simulation” paragraph in the Methods section as below:*

*“Finally, doublets were simulated by merging randomly chosen 3% barcodes with another 3% without overlapping in the matrix. This was repeated for every single read in the BAM file. This simulation modeled the number of reads mapped to the reference and alternative alleles directly. In our simulations, there were 61,576,853 reads in the template BAM file for 12,383 cells, which was equivalent to 4,973 rpc.”*

*As to the sequencing depth, our results on different datasets covered a wide range of sequencing depth from the lowest 5,835 rpc in hashtag dataset (table 3), to 14,495 rpc in stromal cell dataset (table 2), to between 16,000 and 22,000 rpc in fibroblast datasets (table 5, Mix2 - Mix5), to 22,757 rpc in PBMC dataset (table 4), and to 86,148 rpc in one of the fibroblast datasets (table 5, Mix1). The good performance even in the relatively sparse hashtag dataset indicated the robustness of the approach for datasets with different sequencing depths. We also downsampled the stromal dataset, which was reported in table 2, to 2,800 rpc to test performance under very low sequencing depth and the result was also acceptable with overall TPR=0.91 and FDR=0.03. We have included this in the result section “scSplit performed similarly well as demuxlet in demultiplexing merged individually-sequenced three stromal samples”.*

- For the last Results section on batch effects, the conclusion was made based on visual comparison. Can the authors come up with a quantitative way to determine how big or small the batch effects are?

*Thanks for the comment! We used an R package called “cluster” to calculate the Silhouette value for different scenarios in figure 3, and we updated the manuscript as below:*

*“We further checked the gene expression profiles of the previously illustrated three individual stromal samples (Figure 1B and Table 2). We plotted Uniform Manifold Approximation and Projection for Dimension Reduction (UMAP) for non-pooled and pooled scenarios with and without normalization. The samples were more separated from each other in non-pooled and non-normalized scenario (Figure 3A), and got less distant for other scenarios including non-pooled but normalized (Figure 3B), pooled and non-normalized (Figure 3C), and pooled and normalized (Figure 3D). We calculated Silhouette values for each of the UMAPs and got 0.28 for Figure 3A, 0.12 for Figure 3B, 0.14 for Figure 3C and 0.19 for Figure 3D. As bigger Silhouette values indicate larger difference between samples, we could say both normalization and pooling could reduce the batch effects between individually sequenced samples. However, by pooling samples together for sequencing could minimize the potential information loss during normalization..”*

**Second round of review**

**Reviewer 1**

The authors have satisfactorily responded to my previous comments.

**Reviewer 2**

The authors have fully addressed all my questions.
